# Parasitism by *Cuscuta chinensis* is associated with changes in leaf functional traits and hyperspectral characteristics of *Eunymus japonicas*

**DOI:** 10.3389/fpls.2024.1372529

**Published:** 2024-07-25

**Authors:** Jiyou Zhu, Yuxuan Liu, Qinze Zhang, Longqin Li, Hongyuan Li

**Affiliations:** ^1^ College of Environmental Science and Engineering, Nankai University, Tianjin, China; ^2^ School of Soil and Water Conservation, Beijing Forestry University, Beijing, China

**Keywords:** leaf functional traits, spectral characteristics, parasitism, evaluation model, *Cuscuta chinensis*

## Abstract

*Cuscuta chinensis* have a significant regulatory effect on plant growth, but the response mechanism of functional traits to the parasitism of *C. chinensis* and the trade-off relationship between traits and hyperspectral characteristics are not clear. We investigated the functional trait response and hyperspectral characteristics of *Euonymus japonicus*, the most common urban hedge plant in China, to the parasitism of *C. chinensis*. The results showed that the parasitism of *C. chinensis* led to the difference of leaf functional traits: the leaf thickness, stomatal density, and leaf dry matter content were significantly increased, whereas the leaf area, leaf weight, specific leaf area, chlorophyll content index, and leaf tissue density were significantly decreased. Notably, the parasitism of *C. chinensis* changed the spatial distribution pattern of stomata and promoted the stomata to be evenly distributed. Furthermore, the spectral reflectance of leaves treated with the parasitism of *C. chinensis* tended to increase. The parasitism of *C. chinensis* led to the "blue shift" of hyperspectral reflectance of leaves. There was a significant correlation between spectral parameters and leaf functional traits, and leaf biomass accounted for 83% of the variation in reflectance of the water stress band. In general, the parasitism of *C. chinensis* determines the strategic way of plant utilization of resources and affects the change of plant strategy by affecting the difference of traits. Urban plants were more inclined to invest resources in nutrient storage capacity at the expense of resources investment in photosynthetic capacity and defense mechanism. The plant ecological strategy changed from resource acquisition to resource conservation. This finding comes up with a new strategy that urban tree species can modify the plasticity of functional traits for survival and growth under the interference of parasitic plants.

## Highlights

- The parasitism of *Cuscuta chinensis* led to the difference of leaf functional traits.
- The parasitism of *C. chinensis* changed the spatial distribution pattern of stomata and promoted the stomata to be evenly distributed.
- The parasitism of *C. chinensis* led to a "blue shift" of hyperspectral reflectance of leaves.
- There was a close correlation between spectral parameters and leaf functional traits.
- The plant ecological strategy has changed from resource acquisition to resource conservation.

## Introduction

*Cuscuta chinensis* is a kind of parasitic plant which seriously harms urban greening trees. Studies have shown that plant parasitism can significantly affect the growth, reproduction, and biomass distribution pattern of host plants, which eventually leads to changes in the community structure of host plants and seriously threatens the species diversity of local communities (Bouwmeester et al., 2007; Phoenix, 2010; Ogawa et al., 2020). *Euonymus japonicus* Thunb. is one of the most commonly used urban greening trees in China, which has important landscape and ecological value. In recent years, researchers have carried out in-depth research on *E. japonicus*, mainly involving its reproductive characteristics, construction technology, and dust retention ability (Zhu and Xu, 2021). However, there is little research on the ecological strategy of *E. japonicus* to parasitic plants.

There is often a trade-off between the growth and defense of plants (Fernandez et al., 2016). When plants are attacked by biological factors such as herbivores and pathogens, or stressed by abiotic factors such as cold, high temperature, and drought, they can strengthen their defense against abiotic and biotic factors by reducing growth investment and increasing the accumulation of secondary metabolites (Eck et al., 2001; Adriana and Ågren, 2014). The response and adaptation of plants to habitat change has always been one of the hot issues in ecological research. Plant functional traits are the internal physiological structure and external morphological characteristics (Bruner et al., 2023; Mens et al., 2023). The changes of plant functional traits caused by these biological and abiotic factors will affect the interaction between plants and other organisms, such as the construction and maintenance of plant–herbivore, plant–pollinator, plant–seed disperser, and plant–parasitic plant interaction networks (Hedenec et al., 2023). Functional traits of plant leaves are important driving forces for mediating plant–pathogen and plant–herbivore interactions; especially physical defense, chemical defense, and compensation of leaves are important factors for regulating the interaction between plants and herbivores. It is of great significance to understand the differences in functional traits of plant leaves and the influencing factors for exploring the interaction between plants and other organisms. However, although the sensitive response of plant functional traits and their correlation with the environment has been verified, the current research is mostly limited to natural ecosystems whereas the research in urban forest ecosystems is still relatively lacking. Especially under the influence of parasitic plants, there are few studies on the changes of plant functional traits and their ecological strategies of host plants.

Based on this, this study explored the response mechanism of functional traits of urban trees to the parasitism of *C. chinensis* and the trade-off relationship between traits and hyperspectral characteristics and studied the relationship between traits and hyperspectral parameters. Through this experiment, we tried to answer the following questions: (1) Do plant functional traits differentiate with the increase of parasitic intensity? (2) Is there functional coordination among plant functional traits under the effect of parasitism? (3) Is there a relationship between hyperspectral parameters of leaves and plant functional traits? By answering the above questions, we further explain the interaction between urban plants and the environment and clarify the ecological strategy of plants to deal with parasitic stress.

## Materials and methods

### Study area

The study site was located in Nanjing, Jiangsu Province, China. *E. japonicus* (attach to Euonymus L), which grows in the same way and suffers from the parasitism of *C. chinensis* (attach to Cuscuta Linn.), was selected on both sides of urban roads (Changjiang Road). Plant samples were selected on both sides of the road, and 60 plants were selected for each treatment. All plant samples are located on the same road, and they are sampled in turn according to the control group and the parasitic group. To avoid the influence of other environmental factors, the influence of tall buildings was avoided when selecting sampling points. It ensures that the sampling area was consistent in soil, light, temperature, and management. *E. japonicus* has a height of 1.4 m and a ground diameter of 1.3 cm. According to the parasitism of *C. chinensis*, the study plot was divided into control group (CK), and from mild to severe, it was divided into four gradients: T1, T2, T3, and T4. The parasitic proportions of *C. chinensis* were T1: 10%–30%, T2: 30%–50%, T3: 50%–80% and T4: more than 80%.

### Hyperspectral determination

In July, 2023, the spectrum of plant leaves was measured by ASD FieldSpec3 (Analytical Spectral Device, Almero, Netherlands). The spectral measurement time is selected from 11:30 to 14:00 noon (the solar altitude angle is greater than 45). The spectral data acquisition steps are as follows: optimizing spectrometer (OPT)→ whiteboard scanning (WR)→ adjusting transmission mode → the probe is perpendicular to 5 cm above the blade surface → saving the value after the reading is stable, and recalibrating once every 5 min. The scanning time interval of the spectrometer is 0.1 s, and the output curve is the automatic average of 10 original spectra (to avoid the influence of environmental light refraction, the collector wears light-colored work clothes during the operation). The first-order differential spectrum obtains the derivative of the spectrum by calculating the difference between continuous spectral data points. First-order differential processing can highlight the slope change or peak information in the spectrum, so it is very useful for identifying and quantitatively analyzing the peak position and shape in the spectrum. By observing and analyzing the derivative spectrum, we can reveal the subtle features, edges, and changing trends in the spectrum and provide information about sample composition, concentration, and reaction. The spectral parameters are shown in **Table 1** (Zhu et al., 2020a).

**Table 1:** Hyperspectral parameters.

Spectral parameters	Definition
Red edge position, REP	The wavelength position corresponding to RES
Red edge slope, RES	The largest first-order derivative value in the red edge (680 nm~750 nm)
Blue edge position, BEP	The wavelength position corresponding to BES
Blue edge slope, BES	The largest first-order derivative value in the blue edge (490 nm~530 nm)
Yellow edge position, YEP	The wavelength position corresponding to YES
Yellow edge slope, YES	The largest first-order derivative value in the yellow edge (560 nm~640 nm)
Red valley position, RVP	The wavelength position corresponding to RRV
Reflectance of red valley, RRV	Minimum reflectance in the wavelength range of 640 nm~700 nm
Green peak position, GPP	The wavelength position corresponding to RGP
Reflectance of green peak, RGP	Maximum reflectance in the wavelength range of 510 nm~580 nm
Reflectance of water stress band, RWSB	Minimum reflectance in the wavelength range of 1,550 nm~1,750 nm
Leaf water index, LWI	R970/R900
Leaf chlorophyll index, LCI	(R850-R710)/(R850-R680)
Simple ratio index, SRI	R706/R809
Photosynthetic reflectance index, PRI	(R570-R531)/(R570+R531)

### Determination of plant functional traits

Samples were collected in July 2023, and 120 seedlings were collected for each treatment, and 6 mature and healthy leaves were collected for each seedling. The fresh leaf weight (LFW) was measured by HQ531 electronic balance (accuracy 0.01 g, Beijing Huaqing Instrument Company, Beijing). In order to avoid water loss, the measurement was completed within 10 min after the leaves were picked. Leaf area (LA) was measured by YMJ-A portable leaf area meter (accuracy 0.01 mm^2^, Shandong Fangke Instrument Co., Ltd., China, Qingdao). Leaf thickness (LT) was measured with a vernier caliper (accuracy 0.01 mm, Shanghai TAJIMA Tool Company, China, Suzhou). The relative content of chlorophyll was measured by portable chlorophyll meter (Opti-Sciences, Tyngsboro, MA, USA). The specific measurement method was as follows: three points were randomly selected from the upper, middle, and lower parts of the leaf for pinch measurement, and the values of the three points were averaged to obtain the final relative content of chlorophyll in the leaf. Then, the fresh leaves were soaked in pure water and placed in the dark environment of a refrigerator at 5°C for 12 h, and the saturated fresh mass (LSFW) of the leaves was measured by an electronic balance. Finally, the leaves were put into an LC-101–0B air drying oven (Lichen Technology Co., Ltd., Shanghai, China), dried at 75°C to constant weight (48 h), and the leaf dry weight (LDM) was measured. The calculation of leaf functional traits were shown in the following Equations (1–5).


(1) LDMC=LDW/LSFW

(2) LV=LT×LA

(3) LTD=LDW/LV

(4) SLA=LA/LDW

(5) SLW=LDW/LA

In the formula, LDMC—leaf dry matter content, g/g; LDW—leaf dry mass, g; LSFW—saturated fresh mass of leaves, g; LV—leaf volume, cm^3^; LT—leaf thickness, cm; LTD—leaf tissue density, g/cm^3^; SLA—specific leaf area, cm^2^/g; LA—leaf area, cm^2^.

Stomatal traits were measured at 06:00~7:30 a.m. on a sunny day. There were 10 healthy and mature leaves randomly selected, and temporary glass slides of stomata were made on the living leaves to truly restore the original state of stomata in this environment. Pore density was produced by the "imprinting method" (Fiorin et al., 2016). Three temporary slides of stomata were made for each blade, and then five fields of view (713.191 μm × 958.115 μm) were randomly selected to collect stomata images after being magnified 40 times by an XSP-50 optical microscope (Jiangnan Optical Co., Ltd., China, Jiangsu, Nanjing). Finally, the stomata density (number·cm^-2^) was measured by eCognition software.

### Analysis of stomatal point pattern

The paired correlation function g(r) and Ripley's K(r) function were adopted. The K(r) function is defined as the ratio of the expected number of points in a circle with any point as the center and R as the radius to the density of points in the sample. In the g(r) function, the circle in the traditional pattern analysis is replaced by a circle. Among them, the g(r) function eliminates the cumulative effect of the K(r) function, which is an important supplement to the K(r) function.

The expression of the g(r) function is as follows (Equation 6):


(6) g(r)=(K'(r))/2πr(r≥0)

Under the assumption of complete spatial randomness, the value of g(r) is above the envelope line, indicating that the population is clustered and distributed on the r scale. The value of g(r) is located between the envelope lines, which indicates that the population presents random distribution on the r scale. The value of g(r) is below the envelope, which indicates that the population is evenly distributed on the r scale.

### Statistical analysis

One-way ANOVA was used to determine the differences of plant functional traits among different environmental factors, so as to describe the changes of functional traits and leaf reflection spectrum of urban trees after parasitism. Pearson correlation analysis was used to evaluate the correlation between plant functional traits and spectral parameters, and soil traits. Principal component analysis (PCA) comprehensively analyzes many leaf traits of different plants and then analyzes the distribution of plants in the leaf economic spectrum and the explanatory power of parasitic intensity to the variation of plant traits. At the same time, we screened out several groups of parameters with high correlation and made linear regression between plant functional traits and spectral parameters to explore their relationship. All the statistical analyses mentioned above were conducted using Origin 2019b software.

## Results

### Effects of the parasitism of *C. chinensis* on plant functional traits

**Figure 1:**
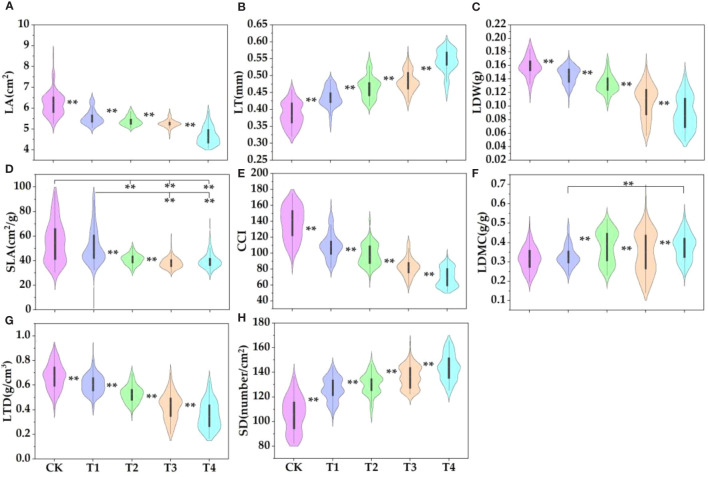
Plant functional traits in different environments. LT, leaf thickness; LA, leaf area; CCI, chlorophyll relative content; SLA, specific leaf area; LDMC, leaf dry matter content; LDW, leaf dry weight (leaf biomass); LTD, leaf tissue density; SD, stomatal density, the same below. The parasitic proportions of *Cuscuta chinensis* were T1: 10%–30%, T2: 30%–50%, T3: 50%–80% and T4: more than 80%. The symbol ** indicates that there is a significant difference between the two indicators.

The parasitism of *C. chinensis* significantly increased the leaf thickness, stomatal density, and dry matter content of *E. japonicus* but significantly decreased the leaf area, leaf biomass, specific leaf area, chlorophyll content, and leaf tissue density (**Figure 1**). Compared with the control group, the leaf area of high-intensity parasitism (T4) and low-intensity parasitism (T1) decreased by 24.9% and 10.8%, respectively (**Figure 1A**). The leaf thickness of T4 and T1 increased by 29.1% and 11.5%, respectively (**Figure 1B**). The leaf biomass of T4 and T1 decreased by 43.6% and 7.8%, respectively (**Figure 1C**). The specific leaf area of T4 and T1 decreased by 28.1% and 7.4%, respectively (**Figure 1D**). The chlorophyll content index of T4 and T1 decreased by 49.1% and 19.9%, respectively (**Figure 1E**). The leaf dry matter content of T4 and T1 increased by 16.2% and 3.4%, respectively (**Figure 1F**). The leaf tissue density of T4 and T1 decreased by 46.6% and 9.3%, respectively (**Figure 1G**). The stomatal density of T4 and T1 increased by 26.4% and 16.0%, respectively (**Figure 1H**).

### Effect of the parasitism of *C. chinensis* on spatial distribution pattern characteristics of stomata in leaves

**Figure 2:**
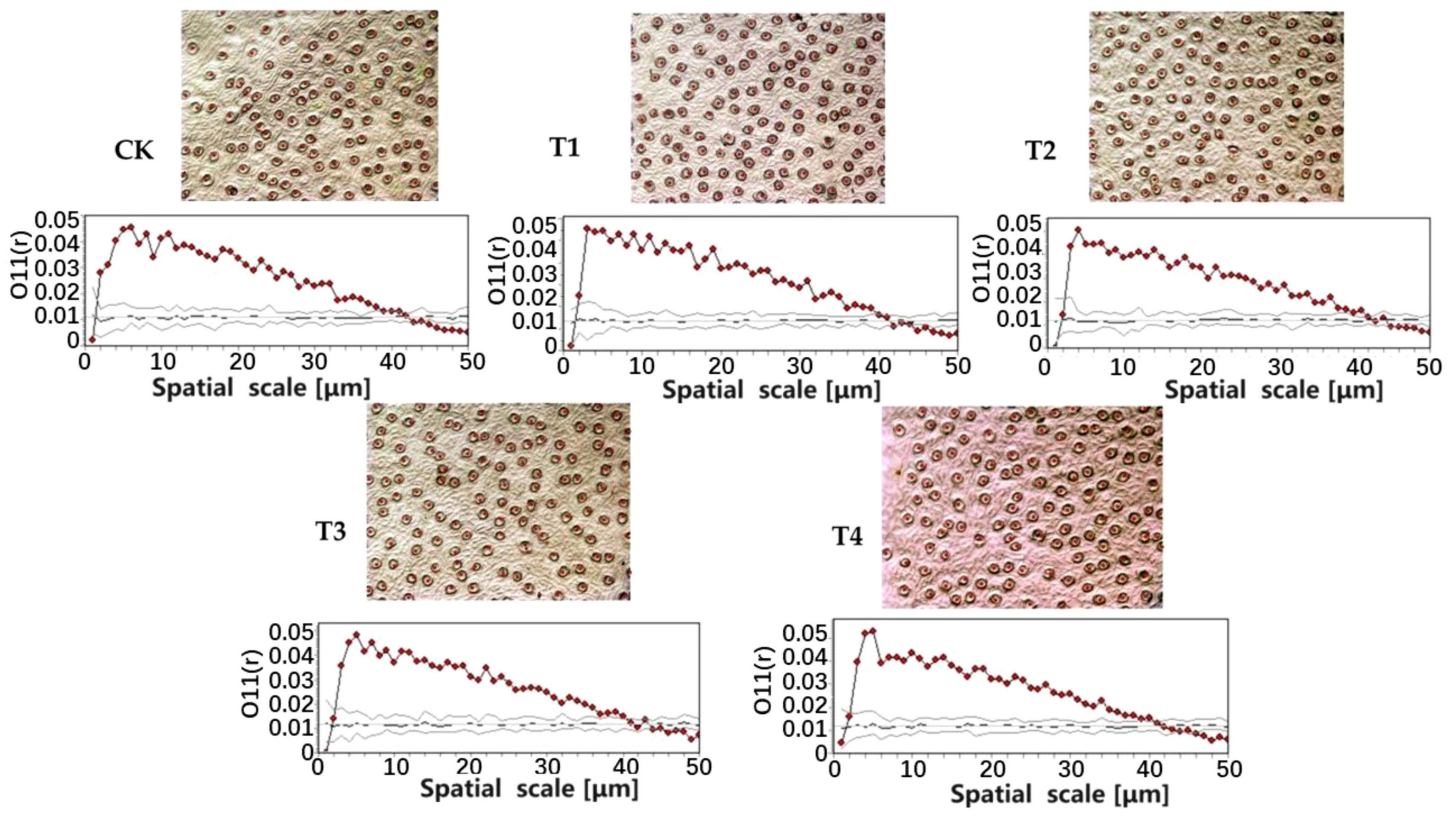
Distribution characteristics of stomata point pattern under different parasitic intensity.

The stomata of *E. japonicus* were aggregated at the scales of 0 μm~39 μm (CK), 0 μm~40 μm (T1), 0 μm~42 μm (T2), 0 μm~41 μm (T3), and 0 μm~41 μm (T4), respectively. It was randomly distributed at the scales of 39 μm~43 μm (CK), 40 μm~44 μm (T1), 42 μm~45 μm (T2), 41 μm~45 μm (T3), and 41 μm~46 μm (T2). It was uniformly distributed at the scales of 43 μm~100 μm (CK), 44 μm~100 μm (T1), 45 μm~100 μm (T2), 45 μm~100 μm (T3), and 46 μm~100 μm (T4). This showed that the parasitism of *C. chinensis* affects the spatial distribution pattern of stomata of *E. japonicus*, which is manifested as follows: with the increase of parasitic intensity, the spatial distribution pattern of stomata of *E. japonicus* becomes more uniform. Plants adapt to the stress of parasitic plants by adjusting the stomatal distribution pattern of leaves; this may be an ecological strategy adopted by urban greening trees to deal with the parasitism of *C. chinensis* (**Figure 2**).

### Response of leaf reflection spectrum to the stress of parasitism of *C. chinensis*

**Figure 3:**
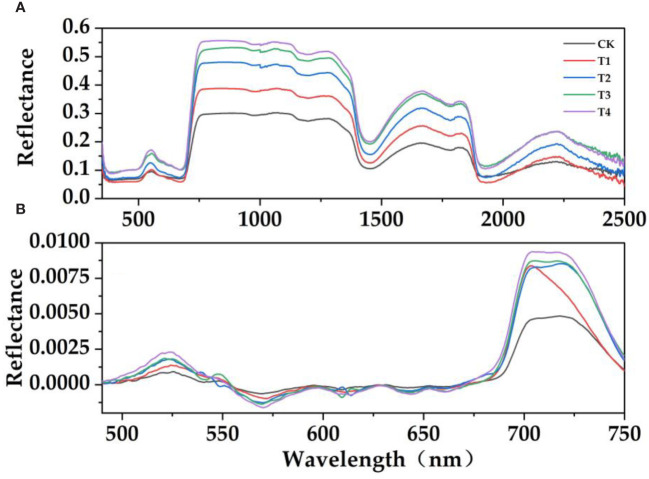
Hyperspectral response characteristics of leaves treated with the parasitism of *C. chinensis*. **(A)** Original reflection spectrum. **(B)** First-order differential spectrum. (The moving average method was used to denoise, and then the interference band was removed and smoothed; it was used as the spectral reflectance characteristic curve of *E. japonicus* leaves).

**Figure 4:**
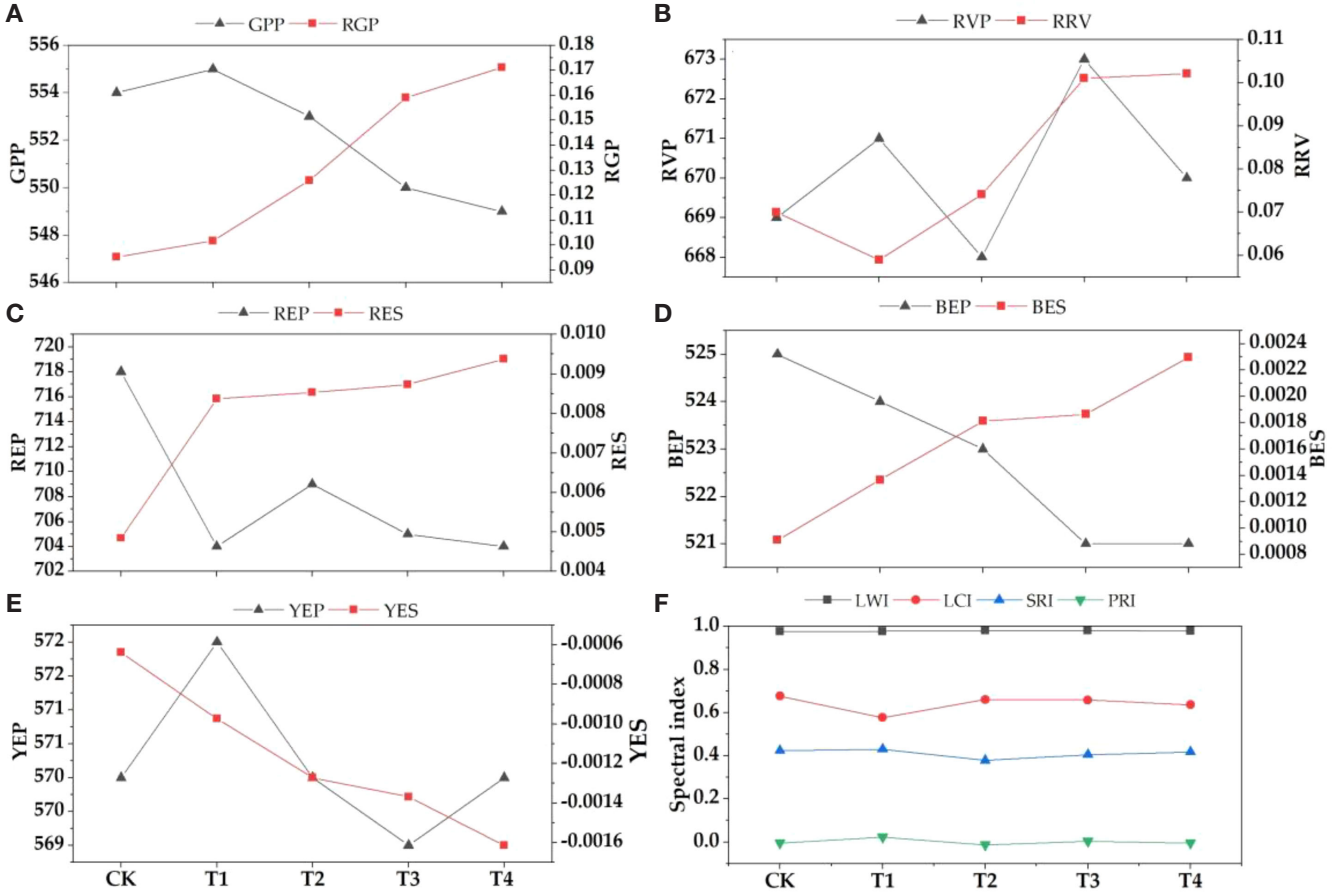
Changes of spectral parameters under different concentrations of parasitism treatment. **(A)** GPP-green peak position, RGP-reflectance of green peak; **(B)** RVP, red valley position, RRV-reflectance of red valley; **(C)** REP-red edge position, RES-red edge slope; **(D)** BEP-blue edge position, BES-blue edge slope; **(E)** YEP-yellow edge position, YES-yellow edge slope; **(F)** LWI-leaf water index, LCI-leaf chlorophyll index, SRI-simple ratio index, PRI-photosyntheticreflectance index.

It can be seen from **Figure 3A** that the spectral reflectance of *E. japonicus* leaves with different parasitic intensity has similar spectral characteristics: there were obvious "green peaks" and "red valleys" in the visible band (390 nm–780 nm). There were reflection peaks near 550 nm in the green band and reflection valleys near 670 nm in the red band. The effects of the parasitism of *C. chinensis* on the spectral characteristics of leaves were different. The parasitism of *C. chinensis* has a significant effect on the leaves of *E. japonicus*, which was mainly reflected in the trend that the spectral reflectance of leaves under parasitism stress was higher than that under natural control. The spectral reflectance of leaves in the visible light band and near-infrared band is higher than that in the control group to some extent (**Figures 3B**, **4**). Under the stress of parasitism, the spectral curve of leaves rises at the green peak and was accompanied by the phenomenon of "blue shift," that is, the center of the green peak shifts to the short-wave direction.

In order to further verify the effect of parasitism on the spectral characteristics of *Buxus microphylla*, the original spectral reflectance of all treatments was first differentiated. With the increase of parasitic intensity, green peak position, red edge position, blue edge position, yellow edge position, and yellow edge slope showed a decreasing trend. The reflectance of green peak, red valley position, reflectance of red valley, red edge slope, and blue edge slope is on the increase. Specifically, the positions of red edge, blue edge, and yellow edge show a decreasing trend, that is, the positions of red edge, yellow edge, and blue edge all move toward the short-wave direction. This means that the parasitism of *C. chinensis* leads to a "blue shift" of hyperspectral reflectance of leaves. The parasitism of *C. chinensis* has little effect on the spectral reflectance index, and the leaf water index has little change, whereas the leaf chlorophyll index and simple ratio index generally show a downward trend.

### Correlation between plant functional traits under the influence of parasitic plants

Leaf area was positively correlated with leaf biomass and specific leaf area and negatively correlated with leaf dry matter content and leaf tissue density. Leaf thickness was negatively correlated with leaf dry matter content. Leaf biomass was positively correlated with leaf dry matter content, leaf tissue density, and stomatal density and negatively correlated with chlorophyll content. Chlorophyll content index was negatively correlated with leaf tissue density. The dry matter content of leaves was positively correlated with leaf tissue density and stomatal density. Leaf tissue density was positively correlated with stomatal density.

### Correlation between spectral parameters and plant functional traits

Leaf thickness was positively correlated with leaf chlorophyll index and negatively correlated with photosynthetic reflectance index, simple ratio index, green peak reflectance of green peak, and red valley reflectance of red valley. Leaf biomass was positively correlated with water stress band reflectivity, red edge slope, and blue edge slope and negatively correlated with yellow edge slope. The chlorophyll content index was negatively correlated with photosynthetic reflectance index photo synthetic reflection index. The leaf dry matter content was positively correlated with red edge slope and blue edge slope. Leaf tissue density was positively correlated with water stress band reflectivity, red edge slope, and blue edge slope and negatively correlated with yellow edge slope (**Table 2**).

**Table 2:** Pearson correlation coefficient between spectral parameters and plant functional traits.

	LWI	LCI	PRI	SRI	RGP	RWSB	RRV	RES	BES	YES
**SA**	0.05	−0.07	0.03	0.13	0.04	0.02	0.11	−0.10	−0.04	0.10
**LT**	−0.01	0.83**	−0.46**	−0.50**	−0.27*	−0.15	−0.31**	−0.13	−0.04	−0.09
**LDW**	−0.17	−0.06	0.01	−0.06	0.14	0.26**	−0.02	0.99**	0.42**	−0.31**
**SLA**	0.09	−0.04	0.07	0.10	0.03	0.07	0.13	−0.07	−0.08	0.15
**CCI**	0.03	0.11	−0.93**	−0.06	−0.15	−0.14	−0.10	−0.13	−0.17	0.07
**LDMC**	−0.04	−0.03	−0.01	−0.08	0.04	0.11	−0.09	0.28**	0.19*	−0.14
**LTD**	−0.15	−0.07	0.04	−0.04	0.16	0.21**	0.01	0.41**	0.33**	−0.29**
**SD**	−0.08	−0.02	0.01	0.02	0.03	0.04	0.03	0.15	0.01	−0.03

"**" means that there is a very significant correlation between traits and spectral parameters, and "*" means that there is a significant correlation between traits and spectral parameters.

### Construction and verification of estimation model of leaf functional traits based on spectral parameters

The spectral parameters with good correlation results and leaf functional traits were respectively used to construct a unitary regression model (**Table 3**). The results showed that the models constructed by leaf chlorophyll index and leaf thickness (R^2^ = 0.684), photosynthetic reflectance index and chlorophyll content (R^2^ = 0.862), and reflectance of water stress band and leaf biomass (R^2^ = 0.826) were the best.

**Table 3:** Univariate linear regression model.

Spectral index	Leaf functional traits	Regression	R^2^
LCI	LT	y = 0.420x + 0.297	**0.684**
SRI	LT	y=−0.275x+0.660	0.253
RGP	LT	y=−0.135x+0.562	0.040
RRV	LT	y=−0.257x+0.565	0.085
PRI	LT	y=−0.789x+0.558	0.215
PRI	CCI	y = −376.860x + 82.899	**0.862**
RWSB	LDW	y=0.758x+0.239	0.068
RES	LDW	y = 4.983x + 0.041	**0.826**
BES	LDW	y=15.494x+0.065	0.126
YES	LDW	y=−18.118x+0.071	0.095
RWSB	LTD	y=0.274x+0.272	0.044
RES	LTD	y=14.976x+0.212	0.164
YES	LTD	y=−77.672x+0.279	0.084
BES	LTD	y=80.870x+0.229	0.177
RES	LDMC	y=6.236x+0.317	0.076
BES	LDMC	y=23.119x+0.342	0.036

Bold words indicate that the two variables have a strong correlation in the linear relationship.

**Figure 5:**
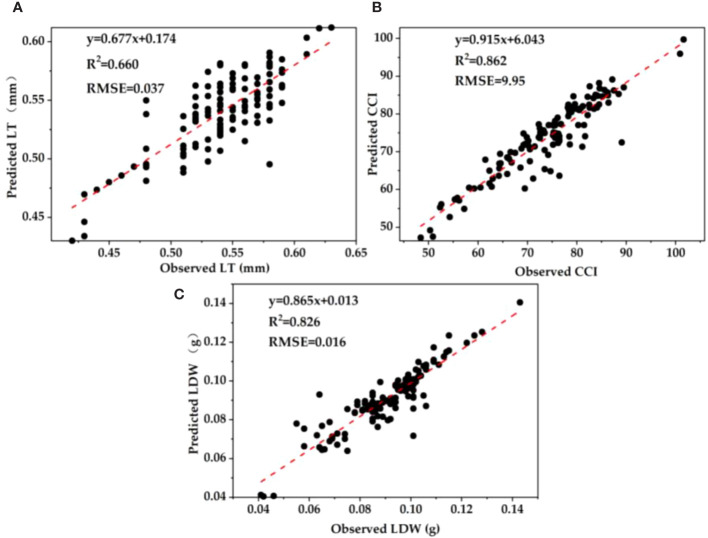
Fitting results of validation set of univariate regression estimation model for leaf functional traits based on spectral parameters. **(A)** LT; **(B)** CCI; **(C)** LDW.

A total of 120 samples were randomly selected to verify the estimation model of plant functional traits with good correlation. The results showed that leaf chlorophyll index and leaf thickness (R^2^ = 0.660, RMSE = 0.037), photosynthetic reflectance index and chlorophyll content index (R^2^ = 0.862, RMSE = 9.95), and reflectance of water stress band and leaf biomass (R^2^ = 0.826, RMSE = 0.016) have the highest accuracy and the smallest error (**Figure 5**). Generally speaking, we can obtain the specific information of plant growth status according to the spectral data of monitoring leaves, especially the plant growth status characterized by the plant functional traits.

## Discussion

### Response of plant functional traits to the parasitism of *C. chinensis*

Many studies have confirmed that parasitic plants have significant effects on plant growth, especially plant height and leaf morphology (Phoenix, 2010). Our results showed that the addition of the parasitism of *C. chinensis* significantly increased the leaf thickness, stomatal density, and leaf dry matter content of *E. japonicus* but significantly decreased the leaf area, leaf biomass, specific leaf area, chlorophyll content, and leaf tissue density. This confirms our first hypothesis. The existing research generally confirmed that leaf area and specific leaf area are closely related to the ability of capturing light resources (Zhang et al., 2022), and the leaf dry matter content is related to the ability of storing nutrients (Pakeman et al., 2011). In this study, the dry matter content of leaves increased with the increase of the parasitism of *C. chinensis*, which indicated that plants showed stronger nutritional retention ability in response to the parasitism of *C. chinensis*. This is similar to the research results of other scholars in grassland ecosystem and desert ecosystem (Pakeman et al., 2011; Blonder et al., 2020). In addition, the parasitism of *C. chinensis* promoted the decrease of leaf tissue density and the increase of leaf thickness. Leaf tissue density and leaf thickness are usually used to characterize the construction level of leaf defense structure (Houter and Pons, 2012; Zhu and Xu, 2021). Therefore, this phenomenon also fully shows that under the action of high-intensity parasitism, plant leaves can reduce the harm of adverse environments by improving their leaf defense level. This strategy was reflected in the trade-off between leaf thickness and leaf tissue density. Stomatal is the circulation window for water vapor exchange between leaves and the external environment, and stomatal density can characterize the water vapor exchange ability of leaves to some extent (Cornic, 2000; Brodribb and Jordan, 2008). The parasitism of *C. chinensis* promotes the increase of stomatal density in leaves, which may be related to the acquisition of water vapor resources in the atmosphere by leaves. In the previous studies, it was also shown that the number of stomata in leaves increased under the stress environment such as drought (Mcadam, 2023), high temperature (Zhu et al., 2020c), and atmospheric particulate pollution (Zhu et al., 2020b). Therefore, the increase of stomatal density may be one of the important ecological strategies for plants to improve water transport ability in response to parasitism stress. It is worth mentioning that this study innovatively found an interesting rule: the parasitism of *C. chinensis* changed the spatial distribution pattern of stomata and promoted the stomata to be evenly distributed in leaves. We suspect that this may be related to the trade-off strategy between plant functional traits. Due to the increase in stomatal density, the total number of windows for water output in plants increases, so adjusting the distribution pattern of stomata is one of the important strategies for plants to reduce water volatilization and dry matter accumulation. Studies have shown that chlorophyll content is closely related to leaf photosynthesis (Zhu and Xu, 2021). The chlorophyll content index decreases with the increase of the city, which shows that plants put more resources into the improvement of leaf defense ability under the stress of parasitism, thus sacrificing the resources for photosynthesis. Leaf area and stomatal area are related to leaf transpiration efficiency to some extent (Li et al., 2021). Therefore, the reduction of these two indexes is to reduce the transpiration area of leaves on the one hand, and to avoid more water loss through stomata on the other hand.

### Trade-off-synergy strategy among plant functional traits

**Figure 6:**
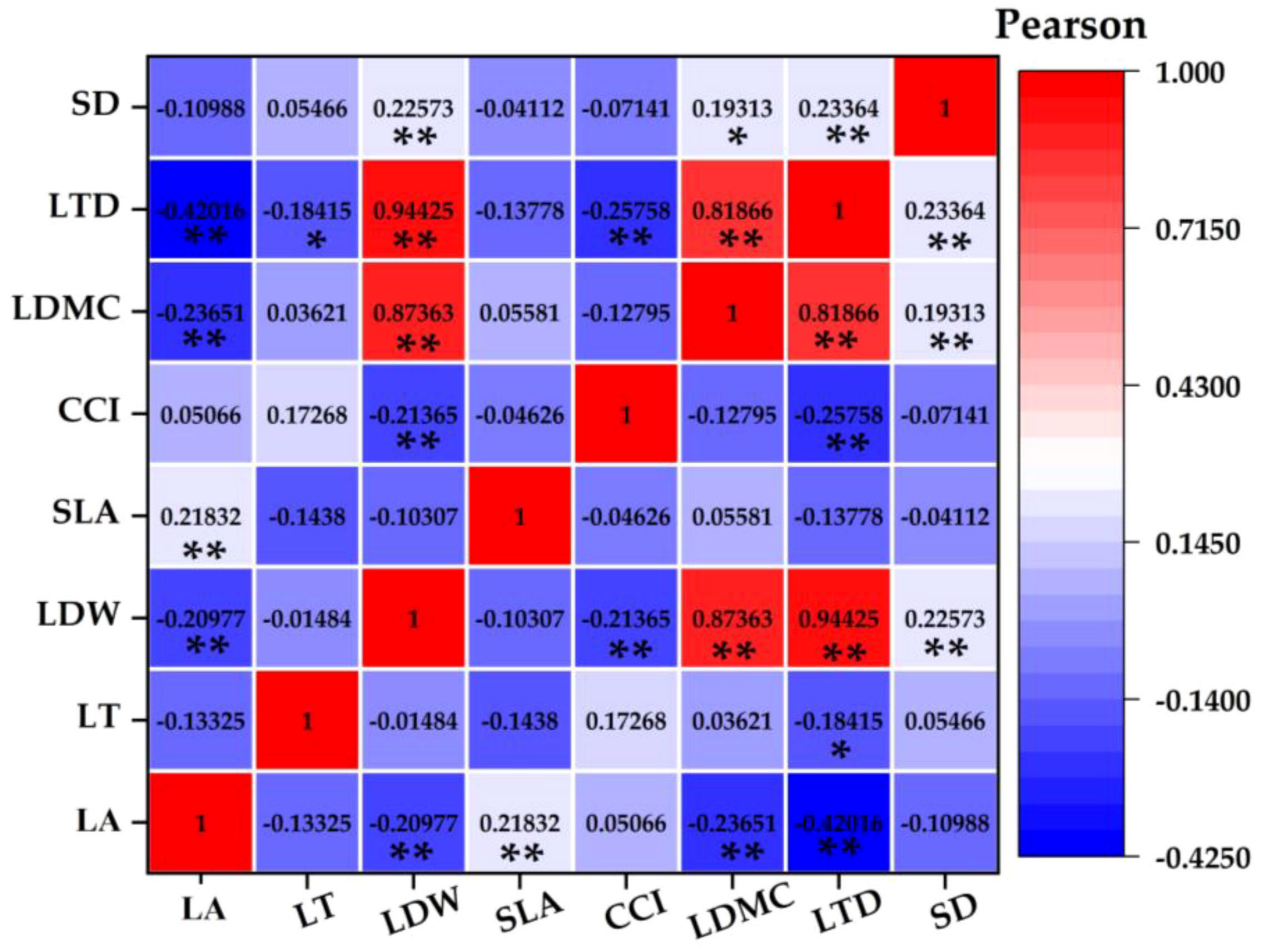
Pearson correlation coefficient between plant functional traits.

**Figure 7:**
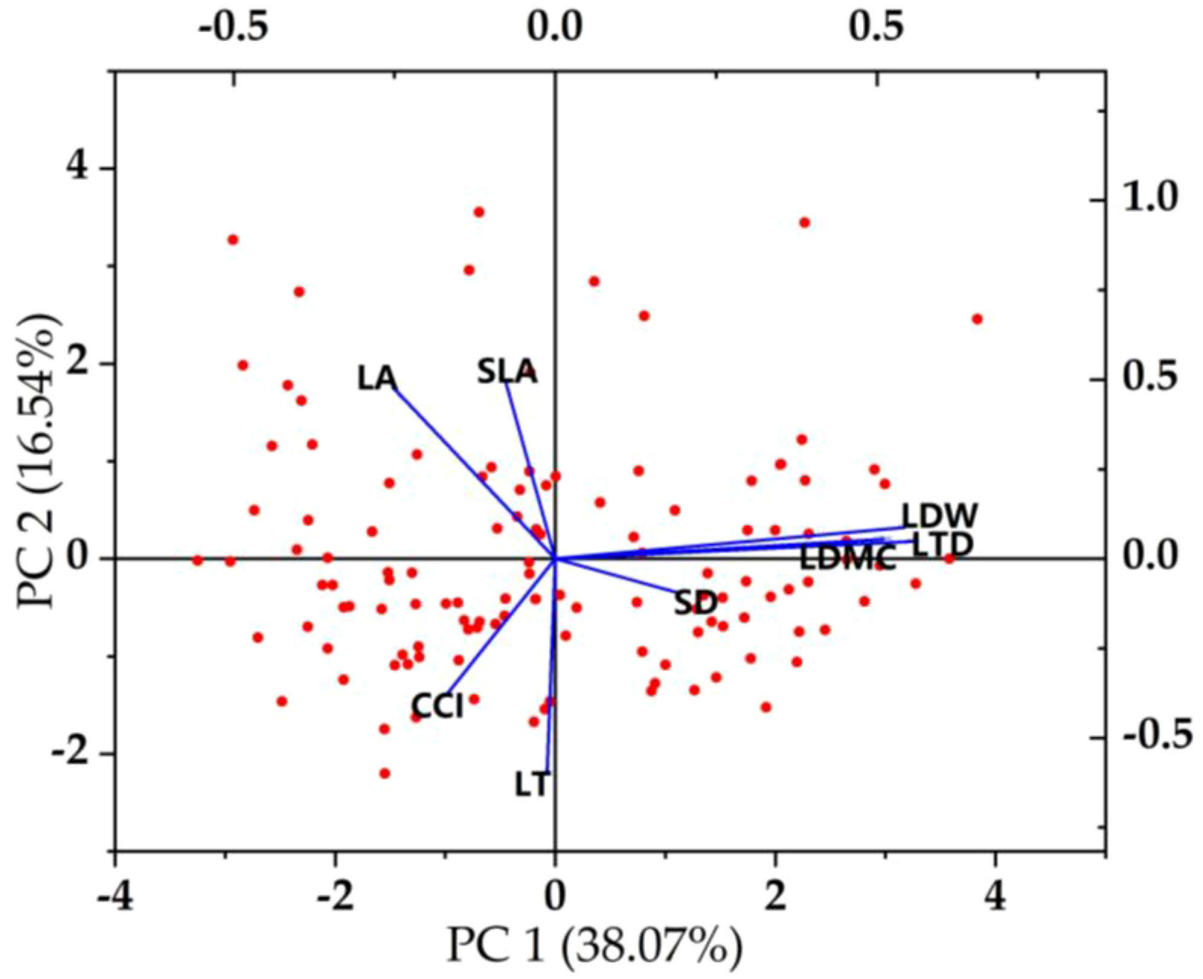
Principal component analysis of plant functional traits.

Plants can adapt to environmental changes through functional adjustment among functional traits and form the best functional combination among traits (Paz et al., 2022; Basile et al., 2023). The content of the parasitism of *C. chinensis* determines the strategic way of plant utilization of resources and affects the change of plant strategy by affecting the difference of traits. This proves our second hypothesis. In this study, there were obvious functional balance changes among the functional traits of *E. japonicus* (**Figures 6**, **7**). For example, leaf area was positively correlated with leaf biomass and specific leaf area and negatively correlated with leaf dry matter content and leaf tissue density. With the increase of the parasitism of *C. chinensis*, the thickness of leaves increased significantly, showing small and thick morphological characteristics as a whole. The leaf dry matter content mainly reflects the ability of plants to preserve nutrient elements (Wakatsuki et al., 2021), and the decrease of leaf area shows that leaves are used for their own nutrient accumulation at the expense of leaf growth resources. This was reflected in the increase of dry matter content of leaves. Chlorophyll content is negatively correlated with leaf tissue density, which is consistent with the research conclusion of Zhu and Xu (2021), and this change was closely related to the self-protection of species under the interference of the parasitism of *C. chinensis*. On the one hand, plants put their own resources into maintaining the storage capacity of nutrients in leaves, which is more conducive to the survival of plants under stress (Zhu et al., 2020a; Zhu and Xu, 2021). On the other hand, because its own resources are relatively limited, resources invested in the accumulation and storage capacity of nutrients will inevitably reduce the input of resources for photosynthetic capacity. Under the parasitism of *C. chinensis*, the leaf area, specific leaf area, and leaf tissue density of plants decreased significantly. In this case, the defensive ability of the leaf itself actually decreases. To maintain its normal growth, more synthetic substances are usually used to increase the storage of nutrients to adapt to the stress environment. The turnover growth rate of leaves with low tissue density is accelerated, and more carbon reserves are used to increase the thickness of leaves. The leaf economics spectrum arranges plants on a specific ecological axis, and one end represents a fast-investment-income strategy with larger leaf area, faster photosynthesis, and respiration rate and shorter leaf life. The other end represents a slow-investment-income strategy that is contrary to the above characteristics. In this study, it was found that the economic spectral axis of the first principal component indicator leaves was strongly correlated with LDMC, LTD, LDW, and SD, which mainly reflected the change of species ecological strategy from resource acquisition to resource conservation.

### Effects of the parasitism of *C. chinensis* stress on spectral characteristics of *E. japonicus* leaves

In visible and near infrared bands, except leaf area, specific leaf area, and stomatal density, the reflection spectra of leaf thickness, leaf dry matter content, leaf biomass, chlorophyll content, and leaf tissue density all respond. This confirms our third hypothesis. In the visible-light band (390 nm–780 nm), it mainly absorbs blue light and red light to provide energy for activities in plants, and the absorption of green light is not as much as that of other visible light, so the reflection spectrum curve forms a "trough" in the blue and red bands and a "peak" in the green band (Yang et al., 2023). The research shows that the red edge position of the near-infrared band of the plant spectrum moves toward the long-wave direction, indicating that the chlorophyll content of the plant leaves increases and moves toward the short-wave direction, indicating that the chlorophyll content of the plant decreases rapidly due to stress (Sims and Gamon, 2002; Louise et al., 2021). This study found that the chlorophyll content decreased rapidly under the stress of the parasitism of *C. chinensis*, which led to the shift of the leaf spectrum in the direction of the short wave at the red edge and the obvious "blue shift" phenomenon. Moreover, the higher the parasitism of *C. chinensis*, the more serious the "blue shift" phenomenon of the red edge position. Another study found that when plants grow vigorously, the red edge shifts red. When the growth declines, the red edge moves blue (Richardson et al., 2021). When plants are affected by the parasitism of *C. chinensis*, their growth is bound to be affected. We believed that this phenomenon was closely related to the changes of plant nutrient transport, carbon and nitrogen metabolism, and chlorophyll content, and such changes are often related to the trade-off relationship of plant functional traits. When no growth inhibitor was added, the chlorophyll content index was high and the color of leaves was dark, so the leaves have strong absorption ability for red light and blue light. With the increase of the parasitism of *C. chinensis*, the nutrients accumulated in the early stage of leaves were invested in different (Wei et al., 2017) functional uses. The chlorophyll content gradually decreases, and the spectral reflectance of green band decreases. Based on the ideas of correlation analysis and regression analysis, this study estimated the functional traits of plants after the parasitism of *C. chinensis*. The results showed that many indexes had good correlation with spectral parameters. After verification with field data, the models based on leaf chlorophyll index and LT, photosynthetic reflectance index and CCI, reflectance of water stress band, and LW show a good fitting relationship between simulated values and observed values, which can be used as the best index to predict plant traits.

## Conclusion

We concluded that the parasitism of *C. chinensis* led to the difference of leaf functional traits and changed the spatial distribution pattern of stomata and promoted the stomata to be evenly distributed. The spectral reflectance of leaves treated with the parasitism of *C. chinensis* tended to increase. The parasitism of *C. chinensis* led to a "blue shift" of hyperspectral reflectance of leaves, which showed that the red edge characteristic was extremely sensitive to parasitic invasion. There was a close correlation between spectral parameters and leaf functional traits, and leaf biomass accounted for 83% of the variation in reflectance of the water stress band. The content of the parasitism of *C. chinensis* determines the strategic way for plants to use resources and influences the change of plant strategies by influencing the difference of traits. Plants were more inclined to invest resources in nutrient storage capacity at the expense of photosynthetic capacity and defense mechanism. The plant ecological strategy changed from resource acquisition to resource conservation. Therefore, the threat of parasitic plants to urban trees should be considered in planting and maintenance. These findings put forward new strategies for tree growth.

## Data availability statement

The original contributions presented in the study are included in the article/supplementary material. Further inquiries can be directed to the corresponding author.

## Author contributions

JZ: Conceptualization, Data curation, Formal analysis, Funding acquisition, Investigation, Methodology, Project administration, Resources, Software, Supervision, Validation, Visualization, Writing – original draft, Writing – review & editing. YL: Data curation, Methodology, Software, Supervision, Writing – original draft. QZ: Conceptualization, Data curation, Investigation, Methodology, Software, Writing – review & editing. LL: Investigation, Software, Writing – original draft. HL: Investigation, Resources, Software, Visualization, Writing – original draft.
